# Breath methane to hydrogen ratio as a surrogate marker of intestinal dysbiosis in head and neck cancer

**DOI:** 10.1038/s41598-020-72115-2

**Published:** 2020-09-14

**Authors:** Nuwan Dharmawardana, Thomas Goddard, Charmaine Woods, David I. Watson, Ross Butler, Eng H. Ooi, Roger Yazbeck

**Affiliations:** 1 Department of Otorhinolaryngology-Head and Neck Surgery, Flinders Medical Centre, Bedford Park, Australia; 2 Discipline of Surgery, College of Medicine and Public Health, Flinders University, Bedford Park, Australia; 3 Department of Respiratory and Sleep Medicine, Women’s and Children’s Hospital, Adelaide, Australia

**Keywords:** Head and neck cancer, Translational research, Cancer, Immunology, Microbiology, Biomarkers, Medical research, Oncology

## Abstract

Exhaled breath compounds can non-invasively detect head and neck squamous cell carcinoma (HNSCC). Here we investigated exhaled compounds related to intestinal bacterial carbohydrate fermentation. Fasting breath samples were collected into 3 litre FlexFoil PLUS bags from patients awaiting a biopsy procedure for suspected HNSCC. Samples were analysed using a Syft selected ion flow-tube mass spectrometer and a Quintron BreathTracker. Two tailed non-parametric significance testing was conducted with corrections for multiple imputations. 74 patients were diagnosed (histological) with HNSCC and 61 patients were benign (controls). The methane to hydrogen ratio was significantly different between cancer and non-cancer controls (p = 0.0440). This ratio increased with tumour stage with a significant difference between T1 and T4 tumours (p = 0.0259). Hydrogen levels were significantly higher in controls who were smokers (p = 0.0129), with no smoking dependent methane changes. There were no differences in short chain fatty acids between groups. Exhaled compounds of intestinal carbohydrate fermentation can detect HNSCC patients. These findings suggest a modified carbohydrate fermentation profile in HNSCC patients that is tumour stage and smoking status dependent.

## Introduction

Over 650,000 new cases of head and neck squamous cell carcinoma (HNSCC) are reported annually worldwide, with poor outcomes despite advances in therapy. Mucosal HNSCC subsites include the nasal cavity, oral cavity, oropharynx, nasopharynx, hypopharynx and the larynx^1^. HNSCC has been described as a ‘lifestyle cancer’ with tobacco smoking and alcohol consumption being the main associated risk factors^2^. However, exposure to high-risk types of human papilloma virus (HPV) have recently been described as a key risk factor for this disease^2^. It has been postulated that local microbial changes in the oral cavity and in the large intestine are associated with changes to the host immunity, driving a systemic inflammation and a pre-disposition towards cancer^3^.

The intestinal microbiome is a complex assemblage of mircoorganisms with direct links to metabolic activity, nutrition, physiology, and immune function. Microbial dysbiosis has been defined as changes in the compositon, load and function of the intestinal microbiome and has been linked to pathologies ranging from gastrointestinal disease to cancer risk and cancer pathogenesis^4^. The effectiveness and adverse event profiles of chemotherapeutic agents have been shown to be dependent upon the intestinal microbiome^5^. Therefore, emergent, non-invasive, technologies that can accurately measure in real-time the fermentation capacity of the large bowel could have future clinical relevance.

The major by-products of microbial carbohydrate fermentation in the large bowel include water, hydrogen, methane and short chain fatty acids (SCFAs) amongst various other gases. Changes in carbohydrate fermentation products in the large bowel have previously been utilised as a surrogate markers of gut microbial dysbiosis^6^. Furthermore, SCFAs produced in the large bowel have been described in vitro studies as protective against head and neck cancers^7^.

Measurement of microbial fermentation products in the large bowel have principally focussed on faecal SCFAs as static markers of microbial function and composition^8^. However, this approach only provides static ‘moment in time’ information on changes to microbial activity. Exhaled breath hydrogen and methane have been previously investigated as non-invasive, real-time surrogate markers of the fermentation status of the large bowel^9^, and changes in these exhaled compounds have been linked to colorectal cancer^10^ and functional gastrointestinal disorders^11^. Breath volatile organic compounds (VOC) have also recently demonstrated promise as non-invasive biomarkers of HNSCC^12^.

Amongst breath VOCs previously identified as discriminatory for HNSCC are SCFAs^13^, suggesting changes to the microbial fermentation patterns in the large bowel of HNSCC patients might be reflected in breath. However, none of the studies that have reported SCFAs as possible HNSCC biomarkers, have determined how breath hydrogen or methane concentrations are modified in HNSCC^14^. Therefore, we aimed to characterise breath excretion profiles of hydrogen, methane and SCFAs in patients with HNSCC. We also aimed to analyse the breath profile relationship to patient characteristics using a large cohort of patients with newly diagnosed cancer and demographically matched controls.

## Patients and methods

### Patients

The HNSCC group was defined as patients with histologically proven SCC of the oral cavity, oropharynx or larynx. These patients underwent biopsy of the primary cancer under general anaesthesia and metastatic sites for histopathological diagnosis as part of a routine diagnostic process. The control group consisted of healthy adult patients who presented for an elective otorhinolaryngology procedure and had no previous history of cancer. Exclusion criteria were as follows: a histological diagnosis of head and neck dysplasia, patients with other concurrent malignancies, patients with head and neck cutaneous malignancies, paediatric patients (age < 18 years) and patients with an active inflammatory condition or infection with or without antibiotic use. We also excluded patients with SCC in neck lymph nodes with no identifiable primary mucosal tumour (Unknown Primary HNSCC). Patients with an oropharyngeal primary lesion had immunohistochemical characterisation of p16 marker, an indirect surrogate (prognostic) marker for human papilloma virus (HPV). Patients with HNSCC were staged using the 8th Edition of the American Joint Committee on Cancer (AJCC) Cancer Staging Manual^15^. Patient history prior to collection of the sample included: smoking status, smoking pack years, alcohol intake (days per week), comorbidities, height, weight, fasting time, tooth brushing, mouth wash use and chewing gum use.

### Breath sample collection

Patients were fasted overnight for a minimum of six hours, as per anaesthetic requirements for elective procedures. Breath samples were collected prospectively in the peri-operative setting at the Flinders Medical Centre and the Royal Adelaide Hospital. All breath samples were collected prior to any surgical procedures or anaesthetic administration. Patients were contacted the day before sample collection and were advised not to wear any perfume or deodorant sprays, not to brush their teeth and not to use any mouth wash in the morning of sample collection to reduce any contamination of breath samples.

On arrival to hospital, patients were asked to rest in a bed for at least fifteen minutes prior to sample collection. During the rest period they answered the questionnaire. They were then asked to take a deep breath in through the nose, followed by a single continuous forced exhalation through the mouth (while pinching their nostrils closed) into a sealed three litre FlexFoil PLUS bag (SKC Ltd, Pennsylvania, USA) resulting in a mixed alveolar gas sample (mixture of alveolar air and respiratory dead space air). A room air sample of the patient environment was also collected immediately after the breath sample collection for comparison and quality assurance. Samples were then transported in a temperature stable container maintained at 37 °C to the laboratory for immediate analysis.

### Sample analysis

#### Hydrogen and methane analysis

30 mL of gas was transferred from the FlexFoil PLUS bag using a Quintron low VOC Luer-Lock syringe with a needle that pierced the septum of the FlexFoil PLUS bag maintaining the isolation from room air. The extracted gas was analysed using the Quintron BreathTracker (Milwaukee, WI, USA) for hydrogen, methane and carbon dioxide levels. The instrument was calibrated with a standard gas mix prior to each run and tested with the standard gas mix periodically for quality assurance.

#### Volatile short chain fatty acid analysis

Remaining breath samples were analysed for SCFA concentrations using Syft selected ion flow tube – mass spectrometry (SIFT-MS). The septum of the FlexFoil PLUS bag was pierced with a non-coring needle and attached directly to the SIFT-MS breath-head (Voice 200, Syft Technologies, Christchurch, NZ). Samples were scanned using the SIFT-MS mass scan mode with three reagent ions (H_3_O^+^, NO^+^, O_2_^+^). Mass spectra were then interrogated using LabSyft software (Version 1.5.1, Syft Technologies, Christchurch, NZ) for acetic acid (2 carbon), benzoic acid (7 carbon), butanoic acid (4 carbon), formic acid (1 carbon) and propanoic acid (3 carbon). All samples were analysed within three hours of collection and were stored in a 37 °C incubator until analyses.

### Statistical analysis

Statistical analysis was performed using IBM SPSS (Version 25. Chicago, IL). Gas concentrations were expressed as median +/− range in parts per million (ppm). Distribution of data was tested using Kolmogorov–Smirnov test and determined to be non-Normal distributions. Therefore, Mann–Whitney-U and Kruskal–Wallis tests were used for two-tailed non-parametric statistical significance testing. Chi-Square statistics were calculated for contingency tables with categorical data. Non-parametric bivariate correlation was conducted using Spearman’s rho correlation coefficients. Statistical significance was considered if p < 0.05. Dunn’s correction factor was applied for multiple comparisons.

### Ethical approval

Ethical approval (HREC reference number HREC/16/SAC/70) was obtained from Southern Adelaide Local Health Network Human Ethics Committee with site specific approvals for Flinders Medical Centre and Royal Adelaide Hospital, Adelaide, South Australia. Informed consent was obtained from participants prior to sample collection. Local and international guidelines were followed as per the Declaration of Helsinki for involving human participants.

## Results

### Distribution of hydrogen, methane and short chain fatty acid in breath across cancer and control patients

Patient groups were matched for age, smoking pack years and fasting time (Table 1). A statistically significant difference in body mass index (BMI) and gender were noted. However, there were no BMI or gender dependent changes in analysed breath compounds (p > 0.05, data not shown).

**Table 1:** Relative differences in patient factors and compounds of interest between cancer and control patient groups.

Patient factors	Controls (n = 61)		Cancer (n = 74)		p value
	Median	Range	Median	Range	
Age (years)	57.00	31–86	56.50	33–88	0.340
BMI (kgm^−2^)	28.26	20–45	26.37	17–38	0.003*
Gender (M:F)	32:29		59:15		0.001*
Smoking pack years	18.00	0–104	29.70	0–295	0.125
Fasting time (hours)	13.00	6–18	12.00	6–26	0.533

Mann Whitney-U test for independent samples and Chi squared testing for contingency tables, *BMI* Body Mass Index, *ppm* parts per million, *Statistical significance (p < 0.05).

**Figure 1:**
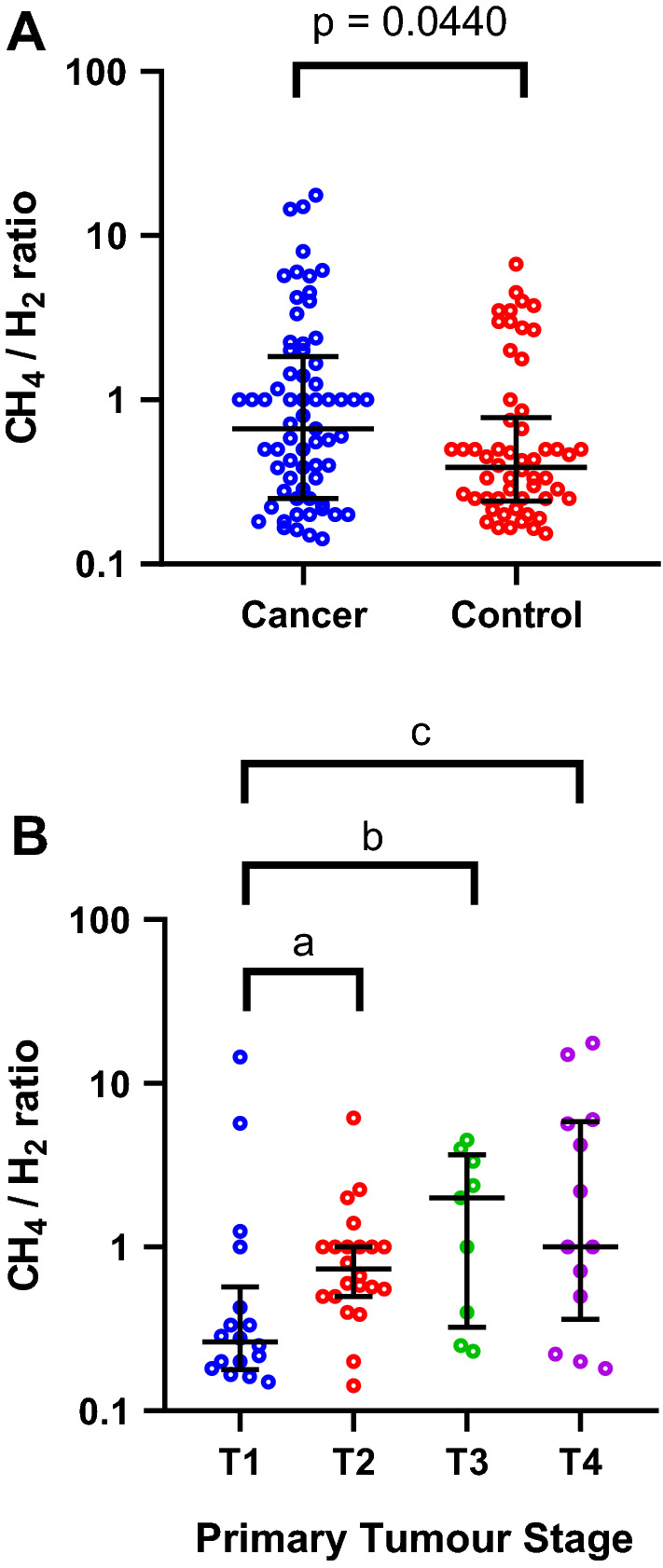
(**A**) Cancer patients compared to control patients (MWU, p = 0.0440), (**B**) T-stage dependent variability in methane to hydrogen ratio. (KW, overall p = 0.0121, Dunn’s correction for multiple comparisons between tumour stage p values; a = 0.1449, b = 0.0687, c = 0.0259). Solid lines describe the median and interquartile ranges. Individual data points indicate the value for each patient.

Intestinal methane, hydrogen and carbon dioxide have a dynamic relationship where high concentrations of methane can significantly affect the local hydrogen concentrations^16^. Therefore, the methane to hydrogen concentration ratio was utilised to explore this dynamic system. The methane to hydrogen ratio was significantly different between patients with (*n* = 74) and without (*n* = 61) cancer (Table 2), with a lower ratio found in the control group compared to cancer (*p* = 0.0440) (Fig. 1A). The CH4:H2 ratio increased with increasing cancer T-stage, and was significantly higher in T4 stage (*Median*: 1.6,* Range*: 0.18–17.48) cancers compared to T1 (*Median: *0.25*, Range: *0–14.5) (p = 0.0259; Fig. 1B). Four volatile SCFAs were detected in exhaled breath using SIFT-MS (Table 2). However, these did not correlate with any patient or tumour factors (MWU, p > 0.05, data not shown).

**Table 2:** Relative differences in compounds of interest between cancer and control patient groups.

Exhaled compounds	Controls (n = 61)		Cancer (n = 74)		p value
	Median	Range	Median	Range	
Hydrogen (ppm)	5.00	0–97	5.00	0–33	0.616
Methane (ppm)	3.00	0–50	5.00	0–58	0.697
CH_4_/H_2_ ratio	0.39	0–6.71	0.67	0–17.67	0.044*
Acetic acid (ppm)	0.31	0.05–6.66	0.28	0.05–4.79	0.620
Butanoic acid (ppm)	0.01	0–0.08	0.01	0–0.13	0.763
Formic acid (ppm)	0.11	0.04–13.40	0.13	0.04–3.14	0.620
Propanoic acid (ppm)	0.02	0–0.12	0.02	0–0.04	0.887

Mann Whitney-U test for independent samples and Chi squared testing for contingency tables, *BMI* Body Mass Index, *ppm* parts per million, *Statistical significance (p < 0.05).

As there have been inconsistencies in the literature for absolute cut-off values to define hydrogen or methane producers^17,18^, we used a range of cut-off levels to separate high and low hydrogen or methane producers and then tested whether they could be used to differentiate between control and cancer patients. There appeared to be a separation between cancer and control patients at a methane concentration of 4 ppm, with more methane producers in the cancer group compared to controls; however, this was not statistically significant. Hydrogen cut-off values between 2 to 5 ppm differentiated cancer and control patients (*p* < 0.05), with a low number of hydrogen producers detected in the cancer group compared to controls.

### Distribution of hydrogen and methane based on HNSCC risk factors and other patient factors

**Figure 2:**
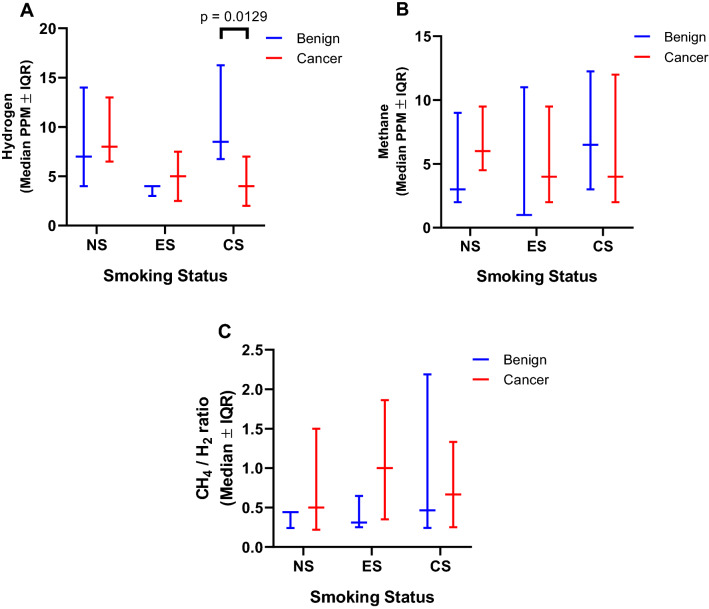
Panel (**A**–**C**) comparing control and cancer patients within each smoking category for hydrogen, methane and their ratio, respectively. *NS* non-smoker, *ES* ex-smoker, *CS* current smoker. P values are indicated where statistical significance is noted. *PPM* parts per million, *IQR* interquartile range. The p-value is only indicated for statistically significant comparisons.

Environmental factors such as tobacco smoking have been previously described as gut microbiome modifiers^19^, and is also well-established as a risk factor for HNSCC. We analysed the effect of smoking on gastrointestinal gases exhaled on the breath. In our dataset, there was no clear relationship between smoking pack years and breath hydrogen or methane concentrations (based on spearman correlation, whole cohort and separated to comparison groups). , When smoking status was categorised to non-smokers (NS), ex-smokers (ES) and current smokers (CS), breath hydrogen levels were 3 ppm higher in NS (*p* = 0.0027) and CS (*p* = 0.0437) compared to ES. However, there was no significant difference in exhaled hydrogen between NS and CS. When these findings were further explored between cancer and control patients, exhaled hydrogen level was significantly lower in the cancer group who were CS compared to the respective controls (Fig. 2A, *p* = 0.0129). There were no significant differences in exhaled methane levels or the methane to hydrogen ratio between smoking groups. There were no differences in methane or the methane to hydrogen ratio between cancer and control (benign) groups when segmented based on smoking status (Fig. 2B,C, *p* > 0.05). Smoking pack year history of current smokers between cancer and control groups were not significantly different (Table 1).

Alcohol intake had no clear effect on breath hydrogen or methane levels. Other patient factors such as their height, weight and fasting time did not have any correlation to breath hydrogen or methane levels in this cohort of patients.

## Discussion

This is the first study to characterise breath hydrogen, methane and volatile SCFAs in a cohort of non-gastrointestinal cancer patients. Using modern breath analysis technologies, our findings identified specific changes in exhaled hydrogen and methane concentrations from patients with cancer that suggest a modified fermentation profile in the large bowel of patients with HNSCC. Furthermore, our results suggest, independent relationships between tumour stage and smoking with gastrointestinal gases exhaled in breath, suggestive of a dynamic and reactive intestinal dysbiosis in HNSCC.

Breath hydrogen and methane are derived almost exclusively from anaerobic microbial fermentation of carbohydrates in the large bowel, and have been used as a non-invasive, surrogate marker of fermentation activity in the colon^18^. Sloan et al. have reported a decrease in breath hydrogen in humans consuming a low Fermentable Oligo-, Di-, Mono-saccharides and Polyols (FODMAP) diet supplemented with oligofructose, finding associations between microbial abundance and hydrogen production^20^. Additionally, the authors reported lower microbial diversity and increased abundance of methanogenic bacteria to be associated with high methane production^20^. Longitudinal studies are needed to quantify exhaled gases of intestinal origin in parallel to quantifying dietary habits, and the composition of the intestinal microbiome, to determine specific systemic effects of intestinal dysbiosis and cancer risk.

Elevated breath methane has been associated with delayed gastrointestinal transit, chronic constipation and diverticulitis^21^. Intestinal methane is produced when CO_2_ is reduced to CH_4_ by methanogenic bacteria using H_2_ as an electron donor. Only three phylotypes of the methanogens have been detected in humans, with the dominant intestinal methanogens being *Methanobrevibacter smithii* (*M. smithii*) and *Methanospaera stadmagnae* respectively^22^. *Methanobreribacter oralis* has also been reported in the oral cavity; however, it’s contribution to overall breath methane is likely negligible^23^. The population distribution of *M. smithii* and other methanogens is variable and can depend on various patient factors such as weight, diet and the presence of pathological conditions^22^. Definitions of a ‘methane producer’ in the literature have ranged from 1 ppm up to 10 ppm methane detected in breath. When we applied the most conservative definition, approximately 28% of patients in our cohort could be classed as methane producers, suggesting a methanogen dominant intestinal microbiome in these individuals^24^. Whilst the North American Consensus Guidelines have recommended 10 ppm breath methane to define a methane producer, it is likely that this value is too restrictive, and methane production should be defined along a concentration spectrum. Fasting breath hydrogen levels are also reported to be dynamic with a variable increase from baseline dependent on a) the carbohydrate ingested and b) the type of gastrointestinal disease^25^. Therefore, clinically the change in breath hydrogen level from baseline is measured over at least two consecutive time points as a standard^26^. The breath hydrogen level is reported to increase between 10 and 20 ppm after carbohydrate ingestion in a normally functioning gastrointestinal tract^25^. Our study did not have the scope to repeat consecutive measurements after ingestion of carbohydrate as this patient cohort were fasting for a surgical procedure. The range of fasted breath hydrogen levels reported in our study are in keeping with other reports^18,27^ with no discernible differences between the cancer and control groups. If breath markers of intestinal gases are to have any utility as a non-invasive measure of the functional capacity of the intestinal microbiome, reliable cut-off thresholds must be established to clearly identify hydrogen and methane producers.

Intestinal dysbiosis has been defined as an imbalance or compositional shift in the gut microbial community that may be associated with disease^21^. Although several studies have described compositional changes of the resident intestinal flora in pathologies ranging from inflammatory bowel disease, cancers and neurological conditions, few, if any, have described the dynamics of microbial fermentation activity. The intestinal hydrogen economy has been previously described as the production and transaction of hydrogen gas produced by hydrogenogens in the colon. Our observation of an increased breath methane to hydrogen ratio in the HNSCC group could be indicative of a modified hydrogen economy in the large bowel, possibly characterised by changes to the composition and activity of methanogenic bacteria. In addition to using hydrogen and CO_2_, methanogens can also derive energy through methanogensis using methanol and formate. Although breath formic acid did not differ between HNSCC and control patient groups, it is possible that the very low concentration of formic acid produced in the large bowel is further diluted when diffused into the blood, or metabolised, likely confounding its accurate measurement in breath. Further studies might consider the application of emerging technologies such as the telemetric capsule to directly measure and quantify intestinal gases^28^. Such an approach could also facilitate real-time information about hydrogen trafficking in the large bowel as a potential surrogate measure of microbial populations.

Our data also indicated a primary tumour stage dependent increase in the methane to hydrogen ratio, more specifically between T1 and T4 that was statistically significant (Fig. 1B). Head and neck cancer T-staging is not exclusively dependent on absolute tumour volume^15^. It is based on several factors a) primary tumour size, b) invasion of anatomy significant for function and locoregional tumour spread and c) depth of invasion (more specific for the oral cavity)^15^. Therefore, the microbial dysbiosis noted in this study as a function of the methane to hydrogen ratio is independent of absolute tumour volume. Although, we acknowledge further studies into absolute tumour volume and its relationship to exhaled hydrogen and methane are warranted.

Periodontitis is a chronic inflammatory disease that affects the supporting structures of the teeth and has been associated with several overlapping risk factors for HNSCC^29^, including poor oral hygiene, smoking and ageing^30^. Although no single causative factor has been identified, *Porphyromonas gingivalis* (*P. gingivalis*) has been isolated as one of the key periodontal pathogens responsible for the onset and progression of disease^31^. Emerging evidence from animal and human studies has suggested that periodontitis and periodontal dysbiosis can lead to oral translocation of the periodontal microbiota, leading to a gastrointestinal dysbiosis^32^. Although we did not directly quantify the incidence of periodontitis, it would be reasonable to hypothesise that a significant portion of our HNSCC patient cohort would have a degree of periodontitis^29^. Therefore, our observed changes to the breath methane to hydrogen ratio could potentially reflect an oral and gastrointestinal dysbiosis. Furthermore, Yang et al.^33^ have recently reported that changes to the oral microbiota were associated with increasing tumour stage. Taken all together, our findings suggest that measurement of breath hydrogen and methane could represent a non-invasive mechanism to monitor microbiome related changes that could be indicative of changes in tumour growth or pathology. Additional, longitudinal population studies, quantifying breath biomarkers to oral and intestinal microbiome analysis are required to accurately determine the relationship of breath biomarkers to periodontal and gastrointestinal dysbiosis and risk for HNSCC.

HNSCC has been described as a ‘lifestyle cancer’, with the typical HNSCC phenotype associated with poor diet, excess alcohol consumption and smoking. These factors have all been previously associated with modifications to the oral and gastrointestinal microbiome. Dietary and lifestyle factors could potentially explain the differences in the methane to hydrogen ratio between HNSCC and control groups. In our study, breath hydrogen was lower in current smokers with HNSCC compared to the controls. Smoking pack year history was not significantly different between current smokers with cancer and the control group, suggesting that the difference in breath hydrogen concentration is unlikely to be independently related to the level of smoking alone. Exhaled hydrogen levels are known to be elevated by smoking if sampled within 2 h of their last cigarette consumption^34^. However, this immediate effect of smoking is unlikely to be relevant to our study population as they abstained from smoking for a minimum of 6 h prior to sampling. Smoking is a known modifier of the intestinal microbiome, and it is likely that our observations are due to a complex, long term interaction between smoking, human microbiome and the tumour dependent metabolic changes^19^. Longitudinal studies are required to determine any relationship between microbial abundance, diversity and microbial activity, as measured by breath hydrogen and methane production. HNSCC generally affects the structure and function of the upper aerodigestive tract, affecting the ability to smell, taste and swallow food^35^. In all head and neck subsites, as the primary tumour size increase (increased T-stage), the patients’ ability to consume certain types of food becomes limited due to structural obstruction and functional loss^36^. Although we collected limited, qualitative dietary information from study participants, it was evident that diets in the cancer group differed from control patients. Therefore, it is likely the profile and amount of food ingested by the cancer patient group was different to the controls, causing downstream changes in the intestinal microbiome. A breath analysis tool that monitors, in real-time, changes to intestinal gases could have utility in dietary and nutritional intervention for cancer patients.

We found no statistically significant relationship between breath SCFAs and HNSCC or patient factors. SCFAs are by-products of bacterial fermentation in the colon, with the most abundant including acetate, propionate and butyrate^37,38^. SCFA production is dependent on a complex relationship between patient fixed factors such as age, dietary fibre intake and other metabolic factors^39^. Previous studies measuring faecal SCFAs have reported contrasting SCFA ratios to what we measured in breath. This is likely due to intermediate metabolic processes following systemic absorption, as well as production of SCFAs via other cell metabolic pathways^40^. Additional controlled studies, using prebiotic substates to stimulate SCFA production are needed to better understand the utility of breath SCFA measurements.

There is a rapid emergence of literature describing an interdependent relationship between the gut microbiome, human immune system and cancer^3^, where intestinal dysbiosis has been implicated in malignant transformation^41^, therapeutic response^42^ as well as long term survival^5^. Breath analysis represents a novel mechanism to non-invasively and assess intestinal fermentation activity that could inform positive intervention. Our findings further establish the utility of human breath as a non-invasive indicator of changes in the gut microbiome and to our knowledge, this is the first reported characterisation of exhaled hydrogen and methane in patients with HNSCC. Further research investigating the direct relationship between exhaled gases, the intestinal and oral microbiome will yield insight into how breath markers of microbial homeostasis could be used to inform personalised treatment plans for this heterogeneous group of patients.

## References

[1] Marur S, Forastiere AA (2016). Head and neck squamous cell carcinoma: Update on epidemiology, diagnosis, and treatment. Mayo Clin. Proc..

[2] Shaw R, Beasley N (2016). Aetiology and risk factors for head and neck cancer: United Kingdom National Multidisciplinary Guidelines. J. Laryngol. Otol..

[3] Li W, Deng Y, Chu Q, Zhang P (2019). Gut microbiome and cancer immunotherapy. Cancer Lett..

[4] Wong SH, Kwong TNY, Wu CY, Yu J (2019). Clinical applications of gut microbiota in cancer biology. Semin. Cancer Biol..

[5] Gopalakrishnan V, Helmink BA, Spencer CN, Reuben A, Wargo JA (2018). The influence of the gut microbiome on cancer, immunity, and cancer immunotherapy. Cancer Cell.

[6] Holma R (2012). Colonic methanogenesis in vivo and in vitro and fecal pH after resection of colorectal cancer and in healthy intact colon. Int. J. Colorectal. Dis..

[7] Krishna S, Brown N, Faller DV, Spanjaard RA (2002). Differential effects of short-chain fatty acids on head and neck squamous carcinoma cells. Laryngoscope.

[8] Pimentel M, Mathur R, Chang C (2013). Gas and the microbiome. Curr. Gastroenterol. Rep..

[9] Peled Y, Weinberg D, Hallak A, Gilat T (1987). Factors affecting methane production in humans. Gastrointestinal diseases and alterations of colonic flora. Dig. Dis. Sci..

[10] Karlin DA, Mastromarino AJ, Jones RD, Stroehlein JR, Lorentz O (1985). Fecal skatole and indole and breath methane and hydrogen in patients with large bowel polyps or cancer. J. Cancer Res. Clin. Oncol..

[11] Perman JA, Modler S, Barr RG, Rosenthal P (1984). Fasting breath hydrogen concentration: Normal values and clinical application. Gastroenterology.

[12] Chandran D (2019). The use of selected ion flow tube-mass spectrometry technology to identify breath volatile organic compounds for the detection of head and neck squamous cell carcinoma: A pilot study. Medicina (Kaunas)..

[13] Shoffel-Havakuk H (2016). Increased number of volatile organic compounds over malignant glottic lesions. Laryngoscope.

[14] Hartwig S (2017). Volatile organic compounds in the breath of oral squamous cell carcinoma patients: A pilot study. Otolaryngol. Head Neck Surg..

[15] Huang SH, Osullivan B (2017). Overview of the 8th edition TNM classification for head and neck cancer. Curr. Treat. Opt. Oncol..

[16] Jang SI (2010). Relationship between intestinal gas and the development of right colonic diverticula. J. Neurogastroenterol. Motil..

[17] Gottlieb K (2017). Selection of a cut-off for high- and low-methane producers using a spot-methane breath test: Results from a large North American dataset of hydrogen, methane and carbon dioxide measurements in breath. Gastroenterol. Rep. (Oxf).

[18] Rezaie A (2017). Hydrogen and methane-based breath testing in gastrointestinal disorders: The North American consensus. Am. J. Gastroenterol..

[19] Savin Z, Kivity S, Yonath H, Yehuda S (2018). Smoking and the intestinal microbiome. Arch. Microbiol..

[20] Sloan TJ (2018). A low FODMAP diet is associated with changes in the microbiota and reduction in breath hydrogen but not colonic volume in healthy subjects. PLoS ONE.

[21] Suri J, Kataria R, Malik Z, Parkman HP, Schey R (2018). Elevated methane levels in small intestinal bacterial overgrowth suggests delayed small bowel and colonic transit. Medicine (Baltimore).

[22] Gaci N, Borrel G, Tottey W, O'Toole PW, Brugere JF (2014). Archaea and the human gut: New beginning of an old story. World J. Gastroenterol..

[23] Carbonero F, Benefiel AC, Gaskins HR (2012). Contributions of the microbial hydrogen economy to colonic homeostasis. Nat. Rev. Gastroenterol. Hepatol..

[24] Levitt MD, Furne JK, Kuskowski M, Ruddy J (2006). Stability of human methanogenic flora over 35 years and a review of insights obtained from breath methane measurements. Clin. Gastroenterol. Hepatol..

[25] Karcher RE, Ruding RM, Stawick LE (1999). Using a cutoff of < 10 ppm for breath hydrogen testing: A review of five years’ experience. Ann. Clin. Lab. Sci..

[26] Rana SV, Malik A (2014). Hydrogen breath tests in gastrointestinal diseases. Indian J. Clin. Biochem..

[27] Tadesse K, Smith D, Eastwod MA (1980). Breath hydrogen (H2) and methane (CH4) excretion patterns in normal man and in clinical practice. Q. J. Exp. Physiol. Cogn. Med. Sci..

[28] Berean KJ (2018). The safety and sensitivity of a telemetric capsule to monitor gastrointestinal hydrogen production in vivo in healthy subjects: A pilot trial comparison to concurrent breath analysis. Aliment Pharmacol. Ther..

[29] Zeng XT (2013). Periodontal disease and risk of head and neck cancer: A meta-analysis of observational studies. PLoS ONE.

[30] Gondivkar SM (2013). Chronic periodontitis and the risk of head and neck squamous cell carcinoma: Facts and figures. Exp. Oncol..

[31] Olsen I, Yilmaz O (2019). Possible role of *Porphyromonas gingivalis* in orodigestive cancers. J. Oral Microbiol..

[32] Olsen I, Yamazaki K (2019). Can oral bacteria affect the microbiome of the gut?. J. Oral Microbiol..

[33] Yang CY (2018). Oral microbiota community dynamics associated with oral squamous cell carcinoma staging. Front. Microbiol..

[34] Tadesse K, Eastwood M (1977). Breath-hydrogen test and smoking. Lancet.

[35] Hammerlid E (1998). Malnutrition and food intake in relation to quality of life in head and neck cancer patients. Head Neck.

[36] Kubrak C (2019). Prevalence and prognostic significance of malnutrition in patients with cancers of the head and neck. Clin. Nutr..

[37] Fernandes J (2013). Age, dietary fiber, breath methane, and fecal short chain fatty acids are interrelated in Archaea-positive humans. J. Nutr..

[38] Yazbeck R, Lindsay RJ, Geier MS, Butler RN, Howarth GS (2019). Prebiotics fructo-, galacto-, and mannan-oligosaccharide do not protect against 5-fluorouracil-induced intestinal mucositis in rats. J. Nutr..

[39] Byrne CS (2018). The effect of L-rhamnose on intestinal transit time, short chain fatty acids and appetite regulation: A pilot human study using combined (13)CO2/H2 breath tests. J. Breath Res..

[40] McNabney SM, Henagan TM (2017). Short chain fatty acids in the colon and peripheral tissues: A focus on butyrate, colon cancer, obesity and insulin resistance. Nutrients.

[41] Russo E, Taddei A, Ringressi MN, Ricci F, Amedei A (2016). The interplay between the microbiome and the adaptive immune response in cancer development. Therap. Adv. Gastroenterol..

[42] Shigematsu Y, Inamura K (2018). Gut microbiome: A key player in cancer immunotherapy. Hepatobiliary Surg. Nutr..

